# Outcomes for high‐risk hepatoblastoma in a resource‐challenged setting

**DOI:** 10.1002/bjs5.50297

**Published:** 2020-05-07

**Authors:** A. Rammohan, M. Rela, G. V. Kumar, J. X. Scott, N. Shanmugam, M. S. Reddy, P. Ramachandran

**Affiliations:** ^1^ Institute of Liver Disease and Transplantation, Sree Balaji Medical College Hospital Bharat Institute of Higher Education and Research Chennai India; ^2^ Ray of Light Foundation, Kanchi Kamakoti Children's Health Institute Laboratory and Diagnostic Services (CHILDS) Trust Hospital CHILDS Trust Medical Research Foundation Chennai India

## Abstract

**Background:**

Outcomes of high‐risk hepatoblastoma have been dismal, especially in resource‐challenged countries where access to chemotherapy and paediatric liver transplantation is limited for the underprivileged. This study aimed to assess the results of treatment of high‐risk hepatoblastoma in a tertiary centre, including patients who had non‐transplant surgical procedures in the form of extended resection.

**Methods:**

A review of patients with high‐risk hepatoblastoma treated between January 2012 and May 2018 was carried out. Perioperative data and long‐term outcomes were analysed.

**Results:**

Of 52 children with hepatoblastoma, 22 were considered to have high‐risk hepatoblastoma (8 girls and 14 boys). The mean(s.d.) age at diagnosis was 35(20) months. Of these 22 children, five died without surgery. Of the remaining 17 who underwent surgery, six had a resection (4 right and 2 left trisectionectomies) and 11 underwent living‐donor liver transplantation. Median follow‐up was 48 (range 12–90) months. Thirteen of the 17 children were alive at last follow‐up and four developed disseminated disease (3 had undergone liver transplantation and 1 liver resection). The overall survival rate at 1, 3 and 5 years was 77, 64 and 62 per cent for the whole cohort with high‐risk hepatoblastoma. In children who had surgery, 1‐, 3‐ and 5‐year survival rates were 91, 82 and 73 per cent for transplantation and 100, 83 and 83 per cent for resection. There was no difference in survival between the two surgical groups.

**Conclusion:**

Excellent results in the treatment of high‐risk hepatoblastoma are possible, even in resource‐challenged countries.

## Graphical abstract

The outcomes of children with high‐risk hepatoblastoma were assessed in the setting of a resource‐challenged nation, where access to organized chemotherapy and liver transplantation remains limited for the underprivileged. Excellent results were demonstrated. Aggressive surgical resection is a successful approach in selected patients with high‐risk hepatoblastoma, who otherwise would be candidates for liver transplantation.

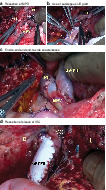

Excellent results in challenging environments

## Abstract (es)

**Antecedentes:**

Los resultados del hepatoblastoma de alto riesgo (*high risk hepatoblastoma*, HRH) han sido pésimos, especialmente en países con recursos limitados, donde el acceso a la quimioterapia y al trasplante hepático pediátrico es limitado para los menos privilegiados. Este estudio tuvo como objetivo evaluar los resultados del HRH en un centro de tercer nivel, incluyendo a los pacientes que se sometieron a procedimientos quirúrgicos diferentes del trasplante en forma de resecciones extendidas.

**Métodos:**

Se realizó una revisión de los pacientes con HRH tratados entre enero del 2012 y mayo de 2018. Se analizaron los datos perioperatorios y los resultados a largo plazo.

**Resultados:**

De 52 niños con hepatoblastomas, 22 fueron considerados HRH (8 pacientes del sexo femenino/14 del sexo masculino). La edad media al diagnóstico fue de 35 ± 20 meses. De estos 22 pacientes, cinco fallecieron sin haber sido intervenidos quirúrgicamente. De los 17 restantes que se sometieron a cirugía, en seis se realizaron resecciones (4 trisectorectomías derechas, 2 trisectorectomías izquierdas) y 11 se sometieron a un trasplante de hígado de donante vivo. La mediana de seguimiento fue de 48 meses (12‐90 meses). Trece de 17 niños estaban vivos en el último seguimiento, y cuatro habían desarrollado enfermedad diseminada (3 habían sido sometidos a trasplante hepático y 1 a resección hepática). La supervivencia global a 1, 3 y 5 años fue del 77,3%, 63,6% y 62% para toda la cohorte de HRH. Entre los que se sometieron a cirugía, las supervivencias a 1, 3 y 5 años fueron del 90,9%, 81,8% y 72,7% para el trasplante y del 100%, 83,3% y 83,3% para la resección. No hubo diferencia en la supervivencia entre los dos grupos sometidos a cirugía.

**Conclusión:**

En países con recursos limitados es posible obtener excelentes resultados en el tratamiento de HRH.

## Introduction

Standardized chemotherapeutic regimens by international groups and refined surgical techniques, including paediatric liver transplantation, have led to significant improvements in treatment outcomes for hepatoblastoma^1^, ^2^. Advances in chemotherapy have enabled more than 60 per cent of tumours initially deemed unresectable to become resectable. Surgery (resection or transplantation) remains the cornerstone of curative treatment.

Based on pretreatment characteristics, hepatoblastomas have been stratified to determine the prognosis and identify the need for more aggressive chemotherapy schedules in the high‐risk variant^2^. Despite this approach, patients with a high‐risk variant have a markedly poorer outcome^2^, ^3^.

Although paediatric tumours are highly treatable, 80 per cent of children with malignancy die because they live in low‐income countries where access to medical care is often inadequate^1^, ^3^. Some of these countries, notably India, have launched cancer initiatives, concentrating treatment resources in regional cancer centres^1^, ^3^, ^4^, ^5^. Government initiatives, non‐governmental organizations and crowd funding have also provided financial support for the treatment of these children. Tertiary paediatric liver transplant centres have achieved outcomes similar to those in Western centres^3^, ^4^, ^5^, ^6^, ^7^, but there are few data on outcomes for hepatoblastoma, and none for high‐risk hepatoblastoma^3^, ^4^, ^5^, ^6^.

For surgically unresectable disease, patients who undergo primary liver transplantation have greatly improved survival compared with those who have a postresection salvage transplant. The International Society of Paediatric Oncology Epithelial Liver Tumour Group (SIOPEL) recommends transplantation on the basis of the pretreatment extent of disease (PRETEXT stages III and IV)^1^, ^2^, ^6^, ^8^, ^9^. Recently, a few studies^10^, ^11^, ^12^, ^13^ have challenged this approach, reporting successful non‐transplant resections in selected patients after chemotherapy, based on the post‐treatment extent of disease (POSTTEXT stages III and IV).

The present study aimed to evaluate the outcome of children with high‐risk hepatoblastoma treated at a tertiary referral centre in a resource‐challenged country where liver transplantation was available. In particular, the study sought to examine outcomes following aggressive liver resection in this subgroup of patients.

## Methods

The study was conducted in accordance with the principles of the Declaration of Helsinki (2008) and good clinical practice guidelines. Following approval from the institutional ethics committee (reference number 002/SBMC/IHEC/2019/1248), review of a prospectively created database of patients with hepatoblastoma managed at the Institute of Liver Disease and Transplantation, Sree Balaji Medical College Hospital, Bharat Institute of Higher Education and Research, Chennai, India, between January 2012 and May 2018 was undertaken. This review was supplemented by data from inpatient and outpatient clinical records, along with subsidiary databases of radiological interventions. Only patients who fulfilled the SIOPEL protocol criteria for high‐risk hepatoblastoma (defined as tumour involving all 4 liver sectors (PRETEXT IV), vascular invasion, extrahepatic disease, α‐fetoprotein (AFP) concentration below 100 ng/ml at diagnosis, or tumour rupture) were included in the analysis. Patients were divided into two groups based on the surgical intervention: group 1 underwent liver transplantation and group 2 had a resection. Outcome variables were analysed both separately and for the whole group, where appropriate.

Diagnosis of hepatoblastoma was based on clinical features and raised serum AFP level. All patients had contrast‐enhanced CT of the chest, abdomen and pelvis, and PRETEXT stage was based on CT findings. Liver biopsy was performed in patients with atypical imaging features and histopathological type identified. Patients were stratified as at standard or high risk according to the SIOPEL guidelines. Treatment decisions were taken by a multidisciplinary team (MDT) and, in line with SIOPEL guidelines, high‐risk hepatoblastoma was treated with cisplatin alternating with a carboplatin–doxorubicin chemotherapy regimen (SuperPLADO)^8^. Serum AFP levels were monitored before each cycle, and imaging was performed after three or four cycles of chemotherapy. Those who had inoperable disease on reassessment were given two further cycles of chemotherapy and reassessed.

Decision regarding liver resection or transplantation was taken after discussion by the MDT. All patients were assessed for likely liver resection, including outflow and inflow vascular reconstructions, with the aim to avoid transplantation where possible. Patients considered to have unresectable disease at postchemotherapy imaging were offered liver transplantation. Donor assessments were completed during the chemotherapy cycles and liver transplantation was performed when an appropriate window was noted after four to six cycles, depending on the response.

In patients undergoing primary transplantation, the donor operation started approximately 1 h before the recipient surgery. In patients undergoing resection, the donor was assessed and kept ready. The protocol was to transfer the donor to the operating room only when tumour was deemed unresectable. Children who were Indian nationals and fulfilled the requisite criteria were added to the deceased‐donor liver transplant waiting list in the hope of benefit, should an organ become available coinciding with the end of chemotherapy.

After surgery (transplant and resection groups), adjuvant chemotherapy was delivered to complete a total of ten cycles (SuperPLADO). During follow‐up, serial AFP levels were measured every 3 months for the first 3 years, every 6 months for the next 5 years, and then annually. Ultrasound imaging of the abdomen was performed every 6 months for the first 3 years of follow‐up, with annual imaging after that. Patients were censored on the date of last follow‐up. Disease‐free survival (DFS) was calculated from the date of initiation of treatment to the date of relapse, progression or death.

### Statistical analysis

Continuous variables are expressed as mean(s.d.) values. Statistical analysis was performed using SPSS® version 20.0 for Windows® (IBM, Armonk, New York, USA). Categorical variables were analysed with the χ^2^ test or Fisher's exact test, as appropriate. Continuous variables were analysed using Student's *t* test or the Mann–Whitney *U* test, as appropriate. The actuarial probability of survival was done by the Kaplan–Meier method, and comparisons were made with the log rank test. *P* < 0·050 was considered statistically significant.

## Results

A total of 52 patients diagnosed with hepatoblastoma were treated during the study period, of whom 22 (8 girls and 14 boys) fulfilled the criteria for high‐risk hepatoblastoma. Two of three patients who fulfilled the high‐risk hepatoblastoma criteria based on low AFP level had PRETEXT IV disease. Five patients fulfilled more than one criterion. Mean(s.d.) age at diagnosis was 35(20) months. Sixteen patients were considered unresectable by standard techniques, including three with extrahepatic disease (*Table* 1). Five children died without surgery; three with progressive disease at POSTTEXT (diffuse lung metastases in 2 and caval involvement extending to the atrium in 1), and two who died before completion of chemotherapy.

**Table 1:** Demographic details of patients with high‐risk hepatoblastoma

	No. of patients (*n* = 22)
**Age (months)** *	35(20)
**Sex ratio (M** : **F)**	14 : 8
**High‐risk hepatoblastoma criteria**	
SIOPEL IV	15
Venous involvement	8
Extrahepatic disease	3
Low AFP level	3
Tumour rupture	0
**Histology**	
Fetal	2
Embryonal/fetal	12
Small cell/poorly differentiated	3
Mixed (epithelial/mesenchymal)	5
**Management**	
Inoperable	5
Resection	6
Liver transplant	11

* Values are mean(s.d.). SIOPEL, International Society of Paediatric Oncology Epithelial Liver Tumour Group; AFP, α‐fetoprotein.

**Figure 1:**
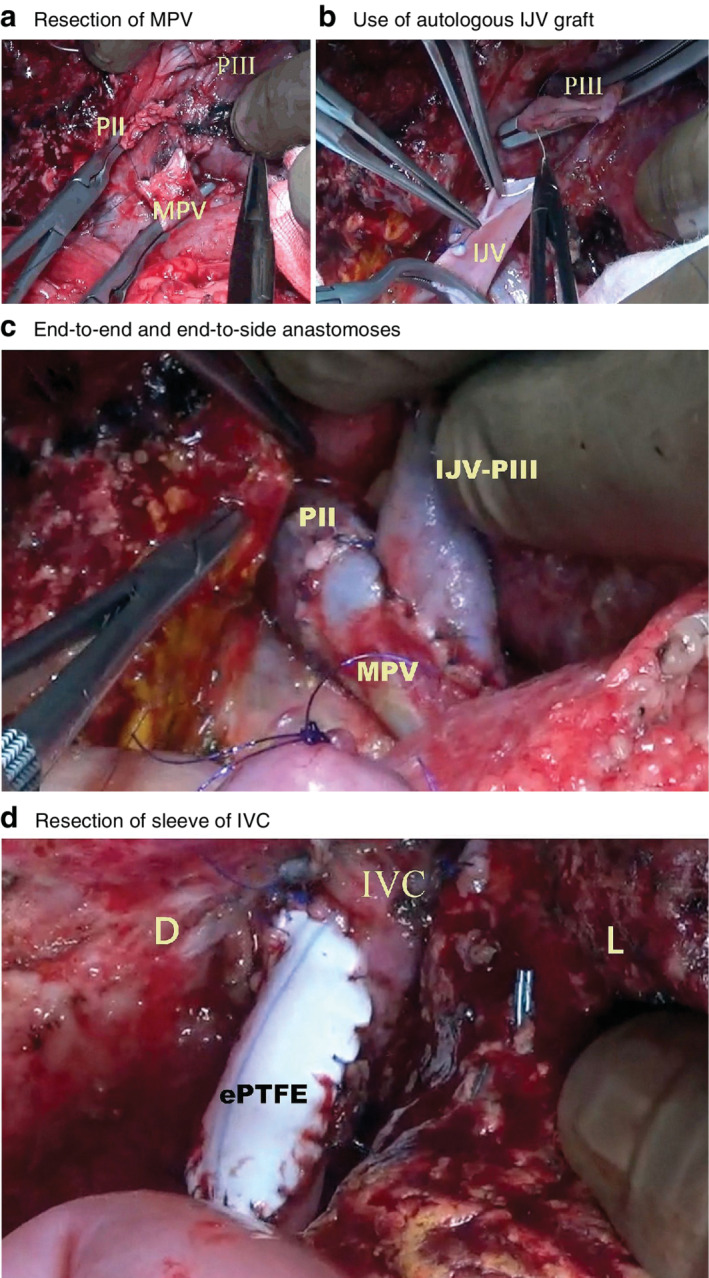
**Vascular resection in high‐risk hepatoblastoma** **a** High‐risk hepatoblastoma with pretreatment extent of disease (PRETEXT) III and vascular invasion after right trisectionectomy with resection of the main portal vein (MPV) and left portal vein. PII, resected end of segment II portal vein (PV); PIII, resected end of segment III PV. **b** Autologous internal jugular vein (IJV) graft used as venous conduit for anastomosis between PIII and MPV. **c** End‐to‐end anastomosis between MPV and PII; end‐to‐side anastomosis between PIII–IJV conduit and MPV. **d** High‐risk hepatoblastoma with PRETEXT IV V3 after right trisectionectomy with resection of a sleeve of inferior vena cava (IVC); closure of IVC with a prosthetic expanded polytetrafluoroethylene (ePTFE) onlay patch. D, diaphragm; L, liver.

Of 17 patients who underwent surgery, six had a resection (4 right and 2 left trisectionectomies) and 11 had transplantation (all left lateral segment grafts from living related donors, with 3 requiring retrohepatic vena cava replacement). No patient selected for resection required conversion to a transplant on the day. One child who had a right trisectionectomy underwent partial excision of the inferior vena cava, requiring an onlay polytetrafluoroethylene patch. Two patients who had a liver resection and three who had a transplant underwent partial resection of the diaphragm to ensure tumour‐free margins. Of these, four were closed primarily and one required prosthetic patch closure. One child with portal vein involvement had a right trisectionectomy with resection of the portal vein and an anastomosis between the main portal vein and segment II/III portal orifices in the Rex recess (*Fig*. 1). Tumour margins were free (resection status R0) in all patients.

No liver failure was seen following resection. Two of the six patients who received a resection had Clavien–Dindo grade II/IIIa complications (bile leak managed conservatively, 1; serous intra‐abdominal collection with right pleural effusion managed by percutaneous drainage, 1). Two patients who underwent living‐donor liver transplantation developed a chyle leak, with a drain fluid triglyceride level above 110 mg/dl; both were managed conservatively by drainage, a fat‐free diet and octreotide, with the condition resolving over a 2‐week period. One patient had acute cellular rejection on liver biopsy that resolved with pulsed steroid therapy, and one developed a polymorphic–polyclonal post‐transplant lymphoproliferative disorder (PTLD) at 15 months after transplantation, managed with immunosuppression reduction. This patient remained in remission at follow‐up 48 months after treatment of PTLD.

**Figure 2:**
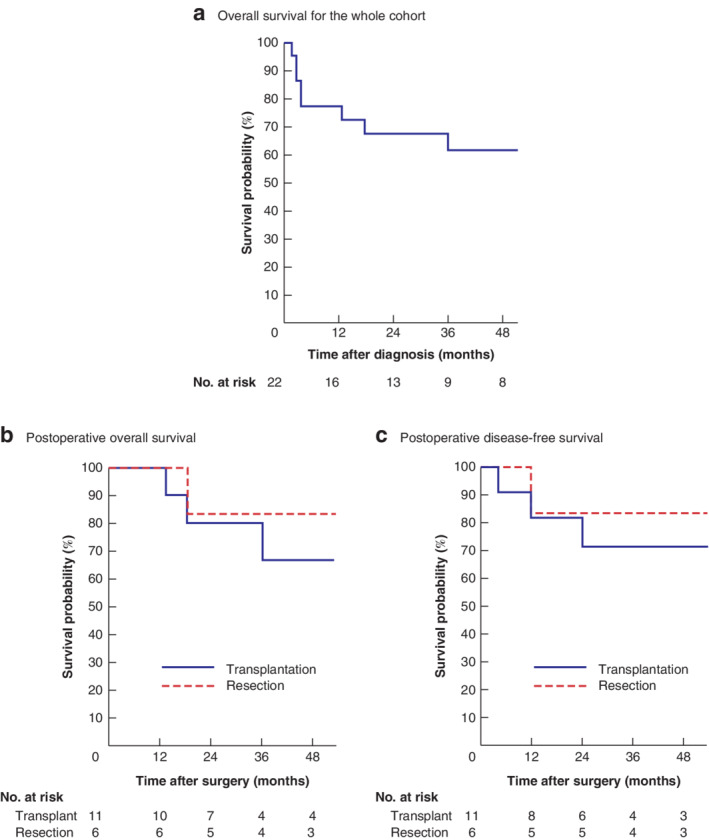
Kaplan–Meier survival curves in patients with high‐risk hepatoblastoma **a** Overall survival in the whole cohort; **b** postoperative overall survival; **c** postoperative disease‐free survival. **b** *P* = 0·663, **c** *P* = 0·627 (log rank test).

Median follow‐up was 48 (range 12–90) months. Lung metastases at diagnosis were identified in four patients; two resolved at POSTTEXT, of which one underwent liver transplantation and the other had a right trisectionectomy. Both remain in remission. Thirteen of the 17 children who had surgery were alive at last follow‐up. Four children developed disseminated disease, three of whom had undergone transplantation and one a resection. The overall survival rate at 1, 3 and 5 years was 77, 64 and 62 per cent respectively for the whole cohort with high‐risk hepatoblastoma. Of the children who underwent surgery, 1‐, 3‐ and 5‐year overall survival rates were 91, 82 and 73 per cent for transplantation and 100, 83 and 83 per cent for resection (*Fig*. 2). There was no difference in survival between the two surgical groups (*P* = 0·663).

## Discussion

Macroscopic residual disease is a known negative prognostic factor in hepatoblastoma. Transplantation eliminates this feature and has therefore become the preferred treatment for high‐risk hepatoblastoma^1^, ^2^, ^6^, ^8^, ^9^. The present experience, however, and as shown by other recent series^10^, ^11^, ^12^, ^13^, indicates that in selected patients extended resection may achieve good long‐term results, even in patients with pulmonary metastases.

In India, living‐donor transplant remains the predominant form of liver transplantation, and most children with high‐risk hepatoblastoma are likely to be offered this treatment^4^, ^5^, ^6^. Nevertheless, several factors have brought non‐transplant resections for these patients back into focus. The long‐term morbidity associated with organ transplantation in patients with hepatoblastoma, the role of long‐term immunosuppression and potential development of secondary malignancy have not been assessed conclusively. Donor morbidity is important^14^, ^15^, ^16^. Despite morbidity remaining low for left lateral segment donation (which forms the majority of grafts in children), there always remains a finite risk of serious complications and mortality. In addition, the financial aspects of liver transplantation, especially in countries without a well developed state‐funded medical system, cannot be neglected^4^, ^7^, ^17^, ^18^. All of these issues, together with the obvious risk of transplantation for the patient and the importance of not exhausting a limited pool of paediatric cadaveric donors, have prompted interest in resectional surgery.

There has been an increasing awareness that some patients with advanced hepatoblastoma may potentially benefit from surgical resection even when, theoretically, they are candidates for primary liver transplantation. Survival rates similar to those obtained after transplantation have been reported in a few series^10^, ^11^, ^12^, ^13^ that employed resection in selected patients with hepatoblastoma. The present series, however, focused specifically on the SIOPEL‐defined subgroup with high‐risk hepatoblastoma, in an environment where transplantation was available as a backup, allowing for more aggressive surgical resections.

Preoperative assessment of patients is crucial when considering non‐transplant surgery. Factors that influenced the team against aggressive resection were: multifocal tumours beyond the borders of anatomical resection; expected liver remnant volumes that were considered too small for adequate postoperative organ function; and unfavourable vascular conditions (either anatomical or functional) as visualized on preoperative imaging. An intraoperative assessment of the tumour burden and the likely state of the functional liver remnant was always the final decision‐maker of the surgical strategy, with transplantation available as backup. Nevertheless, the potential requirement for transplantation highlights the need to manage these children in centres with expertise in both liver surgery and transplantation. It should be acknowledged that the lack of significant difference between the two modalities of surgical treatment may reflect the small number of highly selected patients operated on by a very experienced team.

This experience shows that excellent outcomes can be achieved for high‐risk hepatoblastoma, even in a resource‐challenged setting. Non‐transplant surgical resection can be successful in selected patients and, although technically challenging, is associated with a long‐term oncological outcome that appears comparable to that obtained with primary liver transplantation.

## Disclosure

The authors declare no conflict of interest.
